# The cannabinoid ligands SR141716A and AM251 enhance human and mouse islet function via GPR55-independent signalling

**DOI:** 10.1007/s00018-019-03433-6

**Published:** 2020-01-10

**Authors:** Inmaculada Ruz-Maldonado, Bo Liu, Patricio Atanes, Attilio Pingitore, Guo Cai Huang, Pratik Choudhary, Shanta J. Persaud

**Affiliations:** Department of Diabetes, School of Life Course Sciences, Faculty of Life Sciences & Medicine, King’s College London, London, SE1 1UL UK

**Keywords:** Islets, Cannabinoids, β-Cell function, Insulin secretion, Apoptosis, Proliferation

## Abstract

**Aims:**

Endocannabinoids are lipid mediators involved in the regulation of glucose homeostasis. They interact with the canonical cannabinoid receptors CB_1_ and CB_2_, and it is now apparent that some cannabinoid receptor ligands are also agonists at GPR55. Thus, CB_1_ antagonists such as SR141716A, also known as rimonabant, and AM251 act as GPR55 agonists in some cell types. The complex pharmacological properties of cannabinoids make it difficult to fully identify the relative importance of CB_1_ and GPR55 in the functional effects of SR141716A, and AM251. Here, we determine whether SR141716A and AM251 regulation of mouse and human islet function is through their action as GPR55 agonists.

**Methods:**

Islets isolated from *Gpr55*^+*/*+^ and *Gpr55*^*−/−*^ mice and human donors were incubated in the absence or presence of 10 µM SR141716A or AM251, concentrations that are known to activate GPR55. Insulin secretion, cAMP, IP_1_, apoptosis and β-cell proliferation were quantified by standard techniques.

**Results:**

Our results provide the first evidence that SR141716A and AM251 are not GPR55 agonists in islets, as their effects are maintained in islets isolated from *Gpr55*^*−/−*^ mice. Their signalling through G_q_-coupled cascades to induce insulin secretion and human β-cell proliferation, and protect against apoptosis in vitro*,* indicate that they have direct beneficial effects on islet function.

**Conclusion:**

These observations may be useful in directing development of peripherally restricted novel therapeutics that are structurally related to SR141716A and AM251, and which potentiate glucose-induced insulin secretion and stimulate β-cell proliferation.

**Electronic supplementary material:**

The online version of this article (10.1007/s00018-019-03433-6) contains supplementary material, which is available to authorized users.

## Introduction

The intracellular signalling network that regulates glucose-stimulated insulin secretion from islet β-cells is extraordinarily complex and multifactorial. Insulin secretion is modulated by nutrients, incretin hormones, neurotransmitters and other secreted factors [1], including endocannabinoids. Endocannabinoids are mediators that are synthesised on demand from membrane phospholipids. They can regulate glucose homeostasis through interaction with the canonical cannabinoid (CB) receptors, CB_1_ and CB_2_, and with other cannabinoid-responsive G-protein-coupled receptors (GPCRs), such as G-protein-coupled receptor 55 (GPR55) [2–4]. CB_1_ and GPR55 receptors are abundantly expressed in the hypothalamus, where centers regulating energy homeostasis are located, and peripherally in liver, muscle, adipose tissue, gastrointestinal tract and β-cells [2, 3, 5]. In contrast, although CB_2_ receptors are also present in the central nervous system and endocrine pancreas, they are mainly expressed in cells and organs of the immune system [6], where endocannabinoids mediate immunomodulatory actions.

The role of endocannabinoids in appetite regulation has been extensively studied over the past 20 years [7, 8]. In particular, the CB_1_ receptor was considered to be a promising pharmacological target for weight management due to its activation being associated with hedonic feeding behavior. Rimonabant (SR141716A; Suppl. Fig. S1A) was the first selective antagonist described for CB_1_ receptors in in vitro and in vivo studies [9–11], and it was introduced into clinical use in 2006 as an anti-obesity agent. Rimonabant use was associated with reductions in body weight and waist circumference, and improvements in the profile of metabolic risk factors in patients who were overweight or obese and had atherogenic dyslipidemia [12–15]. Despite being withdrawn due to its adverse psychological effects, almost half of the metabolic benefits, including elevations in circulating adiponectin, occurred independent of weight loss, suggesting direct peripheral effects of this compound [16]. The effects of SR141716A to improve glucose tolerance in obese animal models [17, 18] and humans [19, 20] are likely to have been due, at least in part, to its ability to improve insulin sensitivity, but it is also possible that direct stimulatory effects on islets could contribute to the reductions in blood glucose levels. However, observations of potentiation of glucose-induced insulin secretion by CB_1_ agonists [21, 22] suggest that antagonism of β-cell CB_1_ receptors is unlikely to be responsible for the beneficial effects of SR141716A on glucose homeostasis. It is known that both SR141716A and its iodo analogue, AM251 (Suppl. Fig. S1B), can act as GPR55 agonists in some cell types [23–26]. We have previously reported that AM251 directly stimulated insulin secretion from human islets [21], and a neutral CB_1_ antagonist, LH-21, potentiated insulin release, Ca^2+^ signalling and β-cell survival by acting as a GPR55 agonist in isolated human and mouse islets [4]. It is therefore possible that AM251 and SR141716A have stimulatory effects in islets as GPR55 agonists, rather than CB_1_ antagonists.

In the present study we have therefore evaluated the effects of SR141716A and AM251 on insulin secretion, cAMP and IP_1_ levels, apoptosis and proliferation in human and mouse islets, and we used islets isolated from *Gpr55*^−/−^ mice to determine the requirement for GPR55 in these effects.

## Materials and methods

### Reagents

Culture media and supplements, collagenase type XI, histopaque-1077, DMSO, EDTA, IBMX, carbachol, clonidine, LiCl, exendin-4, forskolin, agarose, bionic buffer and BSA were obtained from Sigma-Aldrich (Dorset, UK). DNeasy Blood and Tissue, RNeasy Mini and QuantiTect SYBR Green PCR kits and qPCR primers for mouse and human CB_1_ (*CNR1*), *GPR119*, *GPR18*, GPR92 (*LPAR5*), *delta-opioid receptor (OPRD1), transient receptor potential cation channel subfamily V member 1 (TRPV1), GPR3, GPR6, GPR12,* and *ACTB* were from Qiagen (Manchester, UK). PCR primers for *Gpr55* genotyping were from Eurofins Genomics (Wolverhampton, UK). SR141716A was from Tocris Bioscience (Abingdon, UK). AM251 and rabbit anti-Ki67 primary antibody were from Abcam (Cambridge, UK). cAMP HiRange and IP-one (IP_1_) assays were from Cisbio (Codolet, France). TaqMan RT-PCR kit, 100 base pairs (bp) DNA ladder, SYBR® DNA gel stain, HEPES, HBSS and DAPI were from Thermo Fisher Scientific (Paisley, UK). Caspase-Glo 3/7 and GoTaq^®^ G2 Green Master Mix were from Promega (Southampton, UK). Recombinant TNFα, IFNγ and IL-1β were from PeproTech EC (London, UK). Guinea pig anti-insulin was obtained from Dako (Cambridge, UK). AlexaFluor 488- and AlexaFluor 594-conjugated secondary antibodies were from Jackson ImmunoResearch Laboratories (Newmarket, UK).

### Animals

A colony of C57BL/6J *Gpr55* homozygous knockout mice (*Gpr55*^*−/−*^) was maintained at King’s College London, with ad libitum access to food and water [3]. Age-matched wild-type (*Gpr55*^+*/*+^) male C57BL/6J mice were purchased from Envigo (Bicester, UK) and maintained in the same conditions as the *Gpr55*^*−/−*^ mice prior to islet isolation. All animal procedures were approved by the King’s College London Ethics Committee and carried out in accordance with the UK Home Office Animals (Scientific Procedures) Act 1986.

### Genotyping

Ear biopsies were removed from weaned mice and DNA samples were prepared using the Qiagen DNeasy Blood and Tissue Kit following the manufacturer’s instructions. DNA was amplified by PCR using 35 cycles with *Gpr55* primers (94 °C: 60 s, 55 °C: 60 s, 72 °C: 60 s; forward: 5′TCTGGATTCATCGACTGTG3′, reverse 1: 5′TCCACAATCAAGCTG3′, reverse 2: 5′GTCACCCATCCAGGTGAT3′. Products were fractionated by gel electrophoresis (150 V, 40 min) using 1.8% agarose in bionic buffer, with predicted amplicons of 207 base pairs for wild-type mice and 299 base pairs for transgenic mice [27].

### Isolation of mouse and human islets

Islets were isolated from 8–12-week-old male *Gpr55*^*−/−*^ C57BL/6J mice and age-matched *Gpr55*^+*/*+^ mice by collagenase digestion of the exocrine pancreas [28], yielding ~ 350 islets per mouse. Human islets used for functional studies and qPCR were isolated from 14 and 3 non-diabetic (Suppl. Table S1), heart-beating pancreas donors at the King’s College Hospital Islet Transplantation Unit with appropriate ethical approval [29]. The average age (± SEM) of the donors for functional studies was 45 ± 2.8 years and the body mass index (BMI) was 28.4 ± 1.3 kg/m^2^, while islets used for qPCR were from donors with average age of 49 ± 4.1 years and BMI of 22.7 ± 1.3 kg/m^2^. Isolated mouse and human islets were maintained in culture overnight (mouse: RPMI-1640; human: CMRL-1066) at 37 °C, 95% air/5% CO_2_ before experimental use [30].

### Dynamic insulin secretion

Groups of 45 mouse or 55 human islets were perifused at a flow rate of 0.5 mL/min with a physiological salt solution [31] supplemented with 2 mM or 20 mM glucose in the absence or presence of compounds of interest using a temperature-controlled perifusion system [30]. Perifusate fractions were collected at 2 min intervals and secreted insulin was quantified by radioimmunoassay [32]. SR141716A and AM251 were dissolved to 10 µM in DMSO, such that the final DMSO concentration was 0.1%, which was also used for control (vehicle) perifusions.

### RNA extraction and quantitative real-time PCR

Total RNA was extracted from groups of 350 *Gpr55*^+*/*+^ or *Gpr55*^*−/−*^ mouse islets or human islets using the Qiagen RNeasy Minikit according to the manufacturer’s instructions and quantified using a NanoDrop spectrophotometer. 500 ng of islet total RNA from mouse and human islets with A_260_/A_280_ ratios between 1.8 and 2.2 were reverse-transcribed into cDNAs using the TaqMan RT-PCR kit. Quantitative real-time PCR (qPCR) using islet cDNAs was performed on a Lightcycler 480 to quantify expression of genes encoding CB_1_, GPR119, GPR18, GPR92, OPRD1, TRPV1, GPR3, GPR6 and GPR12 and levels were normalised to *Actb/ACTB* mRNA expression in the same samples. All GPCR and reference gene primer efficiency (*E*) values were in the range of 1.85–2.15. For all gene quantifications, template cDNAs were diluted in such a way that all quantified genes returned cycle threshold (Ct) values < 30. The relative expression ratio of the targeted genes was calculated based on the *E* and Ct deviation of the employed mouse/human islet preparations, and levels were normalised to *Actb/ACTB* expression in the same samples. Genes expressed < 0.001% of the mean mRNA level of the reference gene used were considered to be present only at trace level, as their expression was less than the lower limit of linear quantification of the QuantiTect primer assays. The primers used for qPCR amplifications are listed in Suppl. Table S2.

### IP_1_ and cyclic AMP accumulation

Groups of five mouse islets or seven human islets were transferred to white-walled 96-well plates in HBSS supplemented with 10 mM HEPES, 0.2% BSA, 5.6 mM glucose and 2 mM IBMX for quantification of cAMP or 50 mM LiCl for assay of IP_1_ levels. For cAMP measurements, islets were incubated for 1 h at room temperature in the absence or presence of 10 µM SR141716A or AM251 using 20 nM exendin-4 as a positive control to induce G_s_ activation. For determination of G_i_ activation, 1 µM forskolin was added to the solutions to stimulate cAMP production so that the inhibitory effect of agents on cAMP generation could be detected. 1 µM of the α_2_ agonist clonidine was used as a control G_i_-coupled ligand. For IP_1_ accumulation, islets were incubated for 1 h at 37 °C in the absence or presence of test agents and 500 µM of the muscarinic agonist carbachol was used as a control G_q_-coupled ligand. Following the subsequent assay steps according to the manufacturer's protocols, islet cAMP or IP_1_ levels were quantified by measuring the fluorescence emission intensity ratio at 665/620 nm using a Pherastar FS microplate reader (BMG Labtech Ltd, Aylesbury, UK).

### Caspase 3/7 activities

Groups of five mouse or human islets were maintained in culture for 24 h in the absence or presence of 10 μM SR141716A or 10 µM AM251, then incubated for a further 20 h in RPMI-1640 with 2% FBS (mouse) or CMRL with 0.2% albumin (human), in the absence or presence of a cytokine cocktail (0.025 U/μL IL-1β, 1 U/μL TNFα, and 1 U/μL IFNγ). Islet cell apoptosis was determined using the Caspase-Glo 3/7 assay [30].

### Islet β-cell proliferation

Groups of 250 mouse or human islets were incubated for 48 h at 37 °C (95% air/5% CO_2_) in RPMI-1640 with 2% FBS (mouse) or CMRL with 0.2% albumin (human), supplemented with 10 μM SR141716A, 10 μM AM251 or vehicle (0.0001% DMSO). Islets were then pelleted at 135 g, fixed with 4% paraformaldehyde and embedded in paraffin. Sections of 5 μm thickness were dewaxed, then antigens were retrieved using citrate buffer (10 mM citric acid, 0.05% Tween 20, pH 6.0). Sections were incubated overnight at 4 °C with primary anti-insulin (guinea pig) and anti-Ki67 (rabbit) antibodies at 1:200 dilution, then incubated with anti-guinea pig AlexaFluor 594 and anti-rabbit AlexaFluor 488 antibodies (1:150 dilution) for 1 h at room temperature. The primary and secondary antibodies are listed in Suppl. Table S3. Images were visualized using a Nikon A1 Inverted Confocal microscope and analysed blindly before quantification using Fiji Image J software (https://fiji.sc) [4]. For each experiment, the images were acquired with the same settings and histological quantifications were performed in paraffin sections that had been immunostained under the same conditions.

### Statistical analyses

Data are shown as mean ± SEM. GraphPad Prism 8.0 (GraphPad Software, Inc.) was used for statistical analyses. Comparisons were analysed by unpaired Student’s *t* test, Wilcoxon signed-rank test and one-way or two-way ANOVA with repeated measures followed by post-hoc tests, as appropriate. *P* < 0.05 was considered statistically significant.

## Results

### SR141716A and AM251 stimulate insulin secretion from human islets

**Fig. 1:**
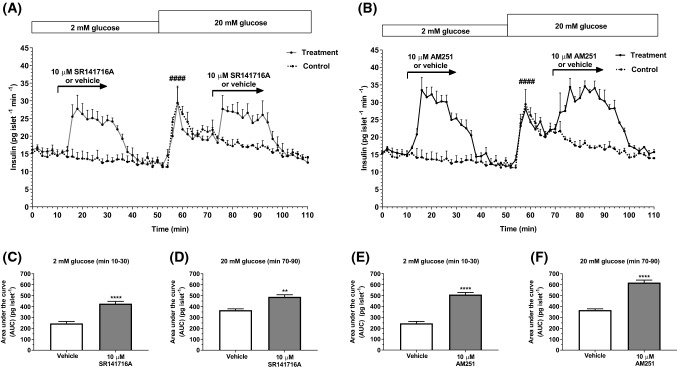
Effects of SR141716A and AM251 on dynamic insulin secretion from human islets. Profiles of insulin secretion from islets isolated from human donors over time (0–110 min) (**a**, **b**) and total insulin AUC (pg islet^−1^) in the absence and presence of SR141716A (**c**, **d**) and 10 μM AM251 (**e**, **f**) at 2 mM glucose (**c**, **e**) and 20 mM glucose (**d**, **f**). 10 μM SR141716A (**a**, **c** and **d**) and 10 μM AM251 (**b**, **e** and **f**) significantly stimulated insulin secretion from human islets at 2 and 20 mM glucose. Data are mean + SEM representative of three separate experiments, each of four replicates, 55 islets per channel. **a**, **b** ^####^*P* < 0.0001 AUC min 50–70 vs. min 0–10; one-way ANOVA, Tukey’s multiple comparisons post test; **c** ****P < 0.0001 AUC min 10–30 vehicle vs. SR141716A; **d** **P < 0.01 AUC min 70–90 vehicle vs. SR141716A; **e** ****P < 0.0001 AUC min 10–30 vehicle vs. AM251; **f** ****P < 0.0001 AUC min 70–90 vehicle vs. AM251; unpaired *t* test

Dynamic perifusions of isolated human islets indicated that SR141716A initiated insulin secretion at 2 mM glucose and it also potentiated glucose-stimulated insulin secretion (Fig. 1a, c and d), and similar stimulatory effects were observed when human islets were exposed to the SR141716A structural analogue, AM251 (Fig. 1b, e and f). These effects on insulin secretion showed a rapid onset and they were readily reversible upon removal of SR141716A or AM251.

### SR141716A and AM251 increase insulin secretion through a GPR55-independent mechanism

**Fig. 2:**
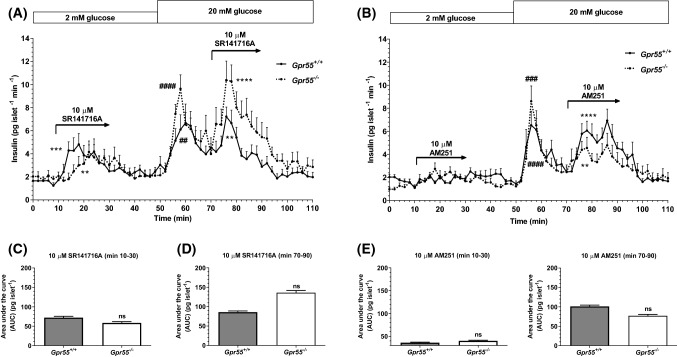
Effects of SR141716A and AM251 on dynamic insulin secretion from mouse islets. Profiles of insulin secretion from islets isolated from *Gpr55*^+*/*+^ (continuous line) and *Gpr55*^*−/−*^ (dashed line) mice over time (0–110 min) (**a**, **b**) and total insulin AUC (pg islet^−1^) of the intervals 10–30 min (**c**, **e**) and 70–90 min (**e**, **f**) between both genotypes. 10 μM SR141716A (**a**) and 10 μM AM251 (**b**) significantly potentiated the plateau phase of glucose-stimulated insulin secretion in islets from both genotypes, with no statistical differences (ns) between the responses (**d**, **f**). 10 μM SR141716A also significantly increased insulin secretion at 2 mM glucose in islets from *Gpr55*^+*/*+^ and *Gpr55*^*−/−*^ mice (**a**). Data are mean + SEM of five independent experiments, each of four replicates, 45 islets per channel. **a** ^##^*P* < 0.01 AUC min 50–70 WT vs. min 0–10 WT, ^####^*P* < 0.0001 AUC min 50–70 KO vs. min 0–10 KO, **P < 0.01, AUC min 70–90 SR141716A WT vs. min 0–10 WT, ****P < 0.0001, AUC min 70–90 SR141716A KO vs. min 0–10 KO; two-way ANOVA, Tukey’s multiple comparisons post test; ***P < 0.001 AUC min 10–30 SR141716A WT vs. min 0–10 WT, **P < 0.01 AUC min 10–30 SR141716A KO vs. min 0–10 KO, unpaired Student’s *t* test. **b** ^###^*P* < 0.001 AUC min 50–70 KO vs. min 0–10 KO, ^####^*P* < 0.0001 AUC min 50–70 WT vs. min 0–10 WT, **P < 0.01, AUC min 70–90 AM251 KO vs*.* min 0–10 KO, ****P < 0.0001, AUC min 70–90 AM251 WT vs. min 0–10 WT; two-way ANOVA, Tukey’s multiple comparisons post test

SR141716A and AM251 were initially classified as selective CB_1_ receptor antagonists/inverse agonists but they also show GPR55 agonist activities in the micromolar range [23, 33] and we have recently reported that another CB_1_ neutral antagonist/inverse agonist, LH-21, stimulated insulin secretion through GPR55-dependent signalling [4]. The ability of SR141716A and AM251 to act via GPR55 in β-cells was investigated by quantifying their effects on insulin secretion from islets isolated from *Gpr55*^+*/*+^ and *Gpr55*^*−/−*^ mice. In these experiments 10 μM SR141716A significantly stimulated insulin secretion at 2 and 20 mM glucose in islets from *Gpr55*^+*/*+^ mice, and it had similar stimulatory effects in islets isolated from *Gpr55*^*−/−*^ mice (Fig. 2a). Calculation of AUC data indicated that there was no statistically significant difference in the responses to 10 μM SR141716A in *Gpr55*^+*/*+^ and *Gpr55*^*−/−*^ islets at either 2 mM or 20 mM glucose (Fig. 2c, d). AM251 (10 μM) also potentiated insulin secretion from islets of both genotypes, but it did not significantly stimulate basal insulin secretion (Fig. 2b). As for SR141716A, the effects of AM251 on insulin secretion were not significantly altered by deletion of GPR55, calculated by AUC (Fig. 2e, f).

### Expression of other islet cannabinoid receptors

**Fig. 3:**
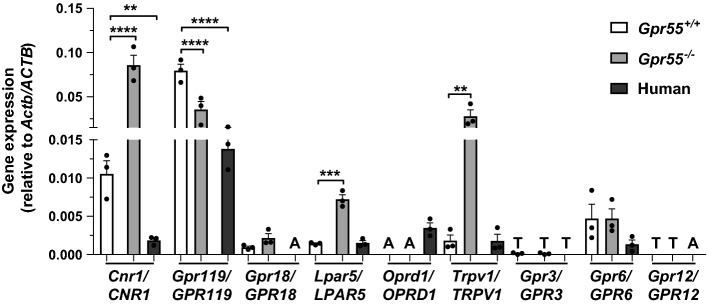
Quantitative RT-PCR of *Cnr1/CNR1*, *Gpr119/GPR119*, *Gpr18/GPR18*, *Lpar5/LPAR5*, *Oprd1/OPRD1, Trpv1/TRPV1*, *Gpr3/GPR3, Gpr6/GPR6, *and* Gpr12/GPR12* mRNA expression in *Gpr55*^+*/*+^ and *Gpr55*^*−/−*^ mouse and human islets relative to *Actb/ACTB* mRNA. **P < 0.01; ***P < 0.001; ****P < 0.0001. Data are expressed as mean + SEM of three non-pooled *Gpr55*^+*/*+^ and *Gpr55*^*−/−*^ mouse islet preparations (350 islets per mouse) and three non-pooled non-diabetic human islet preparations (1000 islets per preparation) and they were analysed by one-way ANOVA. **A**: mRNA absent (i.e., not detected), **T**: trace mRNA expression

In an attempt to identify possible receptors through which SR141716A and AM251 could mediate their functional effects*,* mRNAs encoding the cannabinoid-responsive GPCRs *Cnr1* (CB_1_), *Gpr119, Gpr18, Lpar5* (GPR92), *Oprd1*, *Gpr3, Gpr6 and Gpr12* and the non-selective channel *Trpv1* were quantified by qPCR using cDNA samples from *Gpr55*^+*/*+^ and *Gpr55*^*−/−*^ mouse islets. It can be seen from Fig. 3 that *Cnr1*, *Gpr119, Gpr18, Lpar5, Trpv1 *and *Gpr6* mRNAs were readily detectable in mouse islets, while *Gpr3* and *Gpr12* were expressed at only trace levels and *Oprd1* was not detected. *Lpar5 *and *Trpv1* mRNAs were significantly upregulated in islets isolated from *Gpr55*^*−/−*^ mice, as was expression of *Cnr1* mRNA. Conversely, *Gpr119* mRNA levels in islets were reduced following GPR55 deletion, and there was no significant change in *Gpr18* or *Gpr6* expression. We also quantified expression of these receptors in human islets and found that expression levels of *CNR1* and *GPR119* were significantly lower in human islets than in wildtype mouse islets; *LPAR5* and *TRPV1* expression levels were similar between mouse and human islets, and while *GPR18* and *OPRD1* were present and absent in mouse islets, respectively, the opposite was true in human islets (Fig. 3).

### SR141716A and AM251 do not modulate islet cAMP levels

**Fig. 4:**
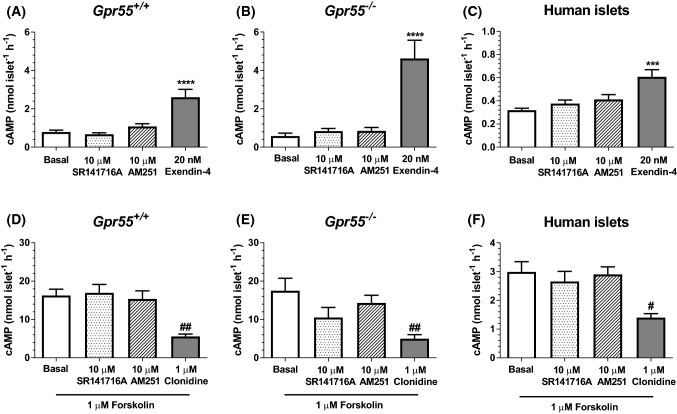
Effects of SR141716A and AM251 on cAMP levels in mouse and human islets. 10 μM SR141716A and AM251 had no effect on basal or forskolin-stimulated cAMP accumulation in islets from *Gpr55*^+*/*+^ (**a**, **d**) and *Gpr55*^*−/−*^ (**b**, **e**) mice or from human donors (**c**, **f**). Nevertheless, 20 nM exendin-4 and 1 μM clonidine significantly increased and decreased cAMP, respectively (**a**–**f**). Data are expressed as mean ± SEM of 4–6 replicates within five individual experiments. ^#^*P* < 0.05 and ^##^*P* < 0.01 clonidine vs. forskolin. ***P < 0.001 and ****P < 0.0001 exendin-4 vs. basal; data were analysed using one-way ANOVA, followed by Dunnett’s multiple comparisons post test

The possibility that the inverse agonist activity of SR141716A and AM251 at islet CB_1_ receptors or their activation of a G_s_-coupled GPCR such as GPR119 could lead to activation of adenylyl cyclase and increase cAMP levels [34] was investigated, to determine if this could explain the stimulatory effects of these ligands on insulin secretion and their independence of signalling via GPR55. However, cAMP quantification indicated that neither ligand had a stimulatory effect on basal or forskolin-stimulated cAMP levels in islets isolated from *Gpr55*^+*/*+^ (Fig. 4a, d) or *Gpr55*^*−/−*^ (Fig. 4b, e) mice, or in human islets (Fig. 4c, f). In these experiments the GLP-1 agonist exendin-4 caused the expected increase in cAMP in both mouse and human islets, and the α_2_-adrenergic agonist clonidine significantly inhibited forskolin-induced elevation in cAMP (Fig. 4).

### SR141716A and AM251 increase islet IP_1_ levels

**Fig. 5:**
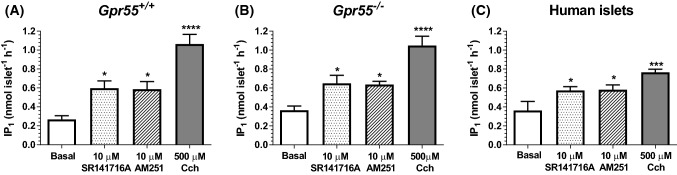
Effects of SR141716A and AM251 on G_q_ signalling in mouse and human islets. **A**–**c** 10 μM SR141716A and AM251 significantly elevated IP_1_ levels in islets from *Gpr55*^+*/*+^ (**a**) and *Gpr55*^*−/−*^ mice (**b**) and human donors (**c**). 500 μM Carbachol (Cch) was used as positive control. Data are expressed as mean ± SEM of three separate experiments, each of 4–6 replicates. *P < 0.05, basal vs. treatment; ***P < 0.001, ****P < 0.0001, basal vs. Cch

As elevations in Ca^2+^ are required for the exocytotic release of insulin and islets express G_q_-coupled putative cannabinoid receptors we investigated the effects of SR141716A and AM251 on G_q_ coupling in islets by quantification of the stable IP_3_ metabolite, IP_1_. Both ligands significantly increased IP_1_ levels in mouse (Fig. 5a) and human (Fig. 5c) islets, as did the muscarinic receptor agonist carbachol (Cch), which signals via G_q_-coupled M_3_ receptors in islets [35]. Consistent with the maintenance of their stimulatory effects on insulin secretion in islets from *Gpr55*^*−/−*^ mice, SR141716A and AM251 also significantly elevated IP_1_ in islets in which GPR55 had been deleted (Fig. 5b).

### SR141716A and AM251 decrease mouse and human islet apoptosis

**Fig. 6:**
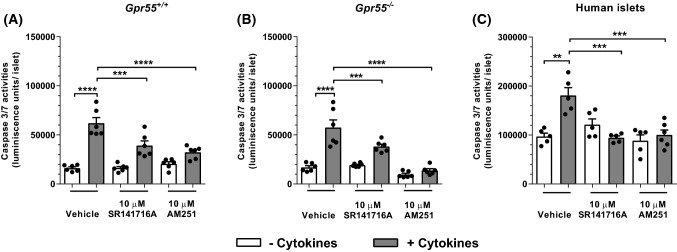
Effects of SR141716A and AM251 on mouse and human islet apoptosis. Effects of 10 μM SR141716A and AM251 on apoptosis of *Gpr55*^+*/*+^ (**a**) and *Gpr55*^*−/−*^ (**b**) islets and human (**c**) islets after 20 h of culture in the absence or presence of a cytokine cocktail (grey bars). Apoptosis was detected by luminescence assay of caspase 3/7 activities. Data are expressed as mean + SEM representative of five independent experiments for both mouse and human islets, each of 6–8 replicates. **P < 0.01, ***P < 0.001, ****P < 0.0001. Data were analysed using one-way ANOVA with repeated measures, followed by Tukey’s multiple comparison post test

Investigation of the effects of SR141716A and AM251 on caspase 3/7 activities in mouse and human islets indicated that both ligands significantly reduced apoptosis induced by 20 h exposure to a cocktail of inflammatory cytokines in islets isolated from *Gpr55*^+*/*+^ mice (Fig. 6a) and these anti-apoptotic effects were also observed in islets from *Gpr55*^*−/−*^mice (Fig. 6b). In addition, SR141716A and AM251 totally blocked cytokine-induced apoptosis in human islets (Fig. 6c). However, although these compounds exerted protective effects against cytokines, they had no effect on basal levels of caspase activity in the absence of cytokines in either mouse or human islets.

### SR141716A and AM251 stimulate human β-cell proliferation

**Fig. 7:**
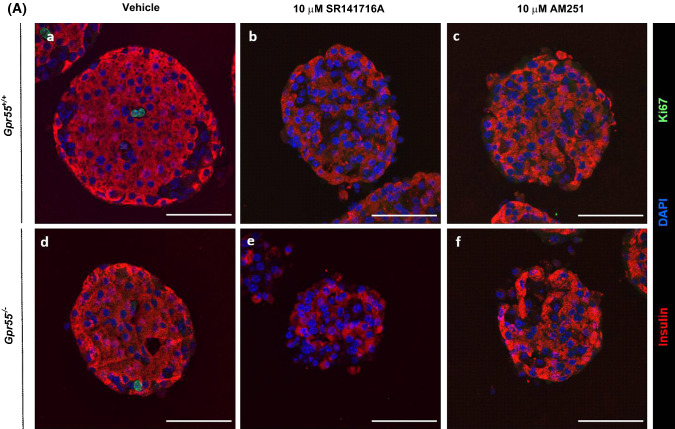

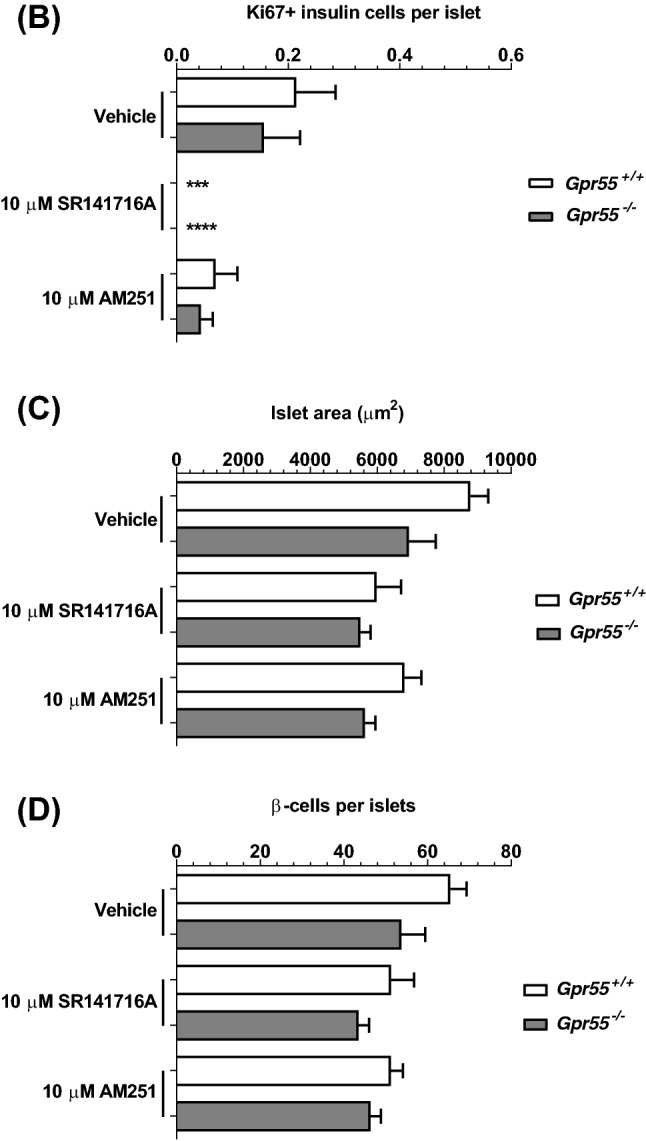
Effects of SR141716A and AM251 on mouse β-cell proliferation. Representative confocal images of paraffin-embedded sections of islets from *Gpr55*^+*/*+^ and *Gpr55*^*−/−*^ mice probed with antibodies directed against insulin (red) and Ki67 (green), and DAPI staining (nuclei; blue) after maintenance of islets in culture for 48 h in the absence or presence of 10 µM SR141716A or AM251 (**a**). Scale bar = 50 μm. Post-acquisition analyses were performed with Fiji Image J software and are shown in **b**–**d**: **b** number of Ki67- and insulin-positive cells per islet; **c** mean islet area (μm^2^) and **d** number of β-cells (insulin-positive cells) per islet. Data were obtained from multiple acquisitions of 47–95 islets per condition, each with a minimum of eight paraffin sections for analysis. *N* = 6 mice per genotype, three independent experiments. ***P < 0.001 and ****P < 0.0001 vs. vehicle *Gpr55*^+*/*+^ or *Gpr55*^*−/−*^. Data were analysed using Wilcoxon signed rank test (**b**) or one-way ANOVA, followed by Dunnett’s multiple comparison post test (**c**, **d**)

**Fig. 8:**
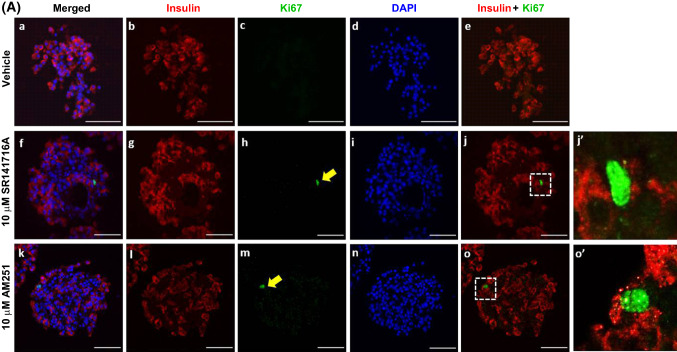

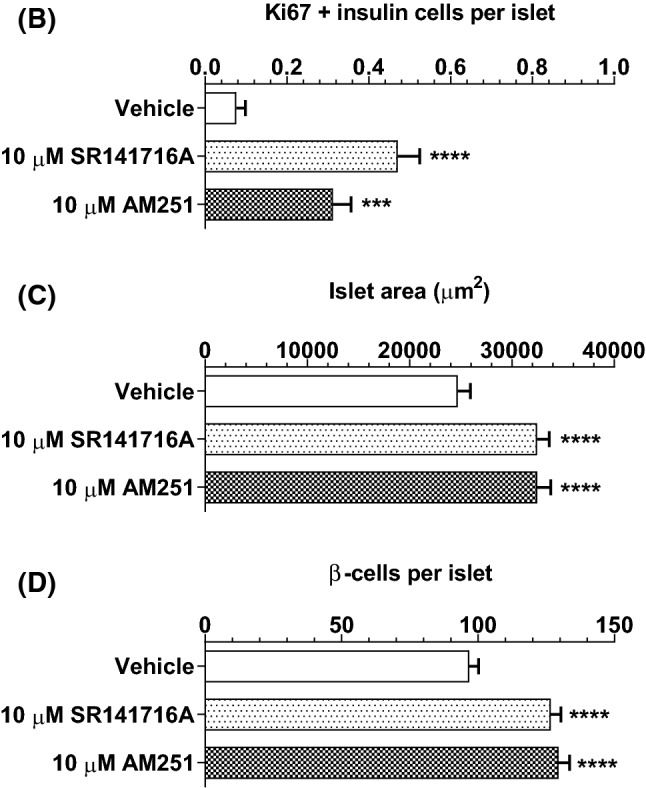
Effects of SR141716A and AM251 on human β-cell proliferation. Representative confocal images of paraffin-embedded sections of human islets probed with antibodies directed against insulin (red) and Ki67 (green), and DAPI staining (nuclei; blue) after maintenance of 250 islets in culture for 48 h in the absence or presence of 10 µM SR141716A or AM251 (**a**). Scale bar = 50 μm. Post-acquisition analyses were performed with Fiji Image J software and are shown in **b**–**d**: **b** number of Ki67- and insulin-positive cells per islet; **c** mean islet area (μm^2^) and **d** number of β-cells (insulin-positive cells) per islet. Data were obtained from multiple acquisitions of 169–210 islets per condition, each with a minimum of eight paraffin sections for analysis from three human donors. ***P < 0.001 and ****P < 0.0001 vs. vehicle. Data were analysed using one-way ANOVA, followed by Dunnett’s multiple comparison post test

Confocal fluorescence immunohistochemistry indicated that the low level of β-cell proliferation in vehicle-treated *Gpr55*^+*/*+^ and *Gpr55*^*−/−*^ mouse islets, identified through co-expression of insulin and the proliferative marker Ki67, was abolished when islets were incubated with 10 μM SR141716A for 48 h (Fig. 7a, b). 10 μM AM251 had similar effects to SR141716A and there was also a trend towards decreased islet area (Fig. 7c) and number of β-cells per islet (Fig. 7d) following 48 h exposure to 10 μM SR141716A or 10 μM AM251. In contrast, exposure of human islets to SR141716A or AM251 for 48 h induced significant increases in the small number of insulin-positive cells expressing Ki67, indicative of increased human β-cell proliferation (Fig. 8a, b). Quantification of human islet confocal images indicated that the ligands also significantly increased islet area (Fig. 8c) and the number of β-cells per islet (Fig. 8d).

## Discussion

The effects of SR141716A on insulin secretion in vitro and in vivo in rodents have been a point of controversy in the literature. Thus, it is reported to decrease insulin hypersecretion in islets isolated from diabetic rats [36] and glucose-induced insulin secretion from mouse islets [37], but another study showed that SR141716A did not significantly affect insulin secretion from mouse islets [38]. Conversely, SR141716A was found to reversibly stimulate insulin secretion from human islets [39], and its chronic administration improved islet function and morphology in diabetic rats [40]. The reasons for discrepancies between different studies are not immediately obvious, but in in vitro experiments with isolated islets stimulatory effects are more likely to be observed in dynamic perifusions [39] rather than in static incubations of islets [37], where potentially inhibitory paracrine mediators such as somatostatin and GABA may accumulate. The effects of the SR141716A analogue, AM251, on insulin secretion are more consistent, with reports that it has insulinotropic effects in mouse islets and BRIN-BD11 cells [41], in βTC6 cells [42] and in human islets [21, 42]. Analysis of the functional effects of SR141716A and AM251 often focus on their classification as CB_1_ receptor antagonists/inverse agonists but they also act as GPR55 agonists in some cell types [23–26], with EC_50_ values of 3.9 μM and 9.6 μM, respectively [33]. Experiments in which 10 mg/kg SR141716A was delivered to mice indicate that it reached 1.9 μg/mL 1 h after i.p administration, equivalent to 4.1 μM in plasma [43], a concentration that is sufficient to induce activity at GPR55 in vivo. GPR55 is expressed by islet β-cells, with its activation enhancing glucose-induced insulin secretion [3–5, 41] so it is possible that the stimulatory effects of SR141716A and AM251 on insulin release could be mediated via their agonist action at β-cell GPR55. Thus, in the current study we investigated the effects of these ligands on insulin secretion, β-cell mass and downstream coupling, and determined whether their effects were dependent on GPR55.

We found that both ligands reversibly stimulated insulin secretion from isolated mouse and human islets, in agreement with earlier reports of direct stimulatory effects of AM251 and SR141716A [21, 39] in perifused human islets. Our observations that SR141716A evoked insulin release at 2 mM glucose are in agreement with the requirement for some rimonabant-treated patients to reduce their anti-diabetic medication [15], and induction of hypoglycaemic episodes by rimonabant in some insulin-treated patients with type 2 diabetes [44]. AM251 also increased insulin secretion from human islets at 2 mM glucose, but was without effect in mouse islets at this sub-stimulatory glucose concentration. These differences in the glucose-dependent effects of AM251 between human and mouse islets may be a consequence of the left-shifted glucose concentration–response profile in human islets [45] or it may reflect species-dependent differences in islet morphology [46] and cannabinoid receptor distribution [2] or arrangement of distinct cannabinoid receptor isoforms within islets [39]. The maintenance of the insulinotropic effects of SR141716A and AM251 in islets isolated from *Gpr55*^*−/−*^ mice demonstrated that their capacity to stimulate insulin secretion is not dependent on GPR55 activation.

The promiscuity in receptor signalling of cannabinoid ligands extends beyond CB_1_/GPR55, and additional GPCRs that are targeted by cannabinoids have been identified, although progress in classification and validation is dependent on identification of the endogenous ligands and development of selective receptor ligands [47]. As SR141716A- and AM251-stimulated insulin secretion was GPR55-independent we investigated the expression of putative islet cannabinoid receptors through which they could act, and determined whether there were alterations in expression in islets in which GPR55 had been deleted. We focused on mRNAs encoding GPR119, GPR92 (*Lpar5*), GPR18, CB_1_, OPRD1 and TRPV1 since they have previously been implicated as targets of cannabinoids [48–51]. In addition, we quantified *Gpr3*, *Gpr6* and *Gpr12* mRNAs because these orphan G_s_-coupled Class A GPCRs have a close phylogenetic relationship with cannabinoid receptors and the phytocannabinoid cannabidiol has recently been identified to act as an inverse agonist at these receptors [52]. We found that in addition to CB_1_ (C*nr1*) mouse islets also expressed mRNAs encoding G_s_-coupled GPR119 and GPR6, G_q_-coupled GPR18 and GPR92 (*Lpar5*), and the non-selective cation channel TRPV1 while mRNA-encoding G_i_-coupled delta-opioid receptor (*Opdr1*) was absent, and *Gpr3* and *Gpr12* mRNAs were only expressed at trace levels. *Cnr1*, *Lpar5* and *Trpv1* were upregulated following GPR55 deletion, while mRNA encoding GPR119 was significantly decreased in *Gpr55*^*−/−*^ islets. To add to the complexity, GPR55 may be able to form heterodimers with CB_1_ receptors and impairment of this following deletion of GPR55 and the consequent upregulation of *Cnr1* in islets could have functional implications for SR141716A and AM251 signalling. However, our previous observations that CB_1_ agonists stimulate insulin secretion [21, 22] are inconsistent with the GPR55-independent effects of SR141716A and AM251 on insulin release being via upregulation of CB_1_ receptors in islets from *Gpr55*^*−/−*^ mice, since these ligands are CB_1_ antagonists.

Quantification of islet cAMP levels indicated that neither ligand affected basal or forskolin-stimulated cAMP production in either *Gpr55*^+*/*+^ or *Gpr55*^*−/−*^ islets, or human islets, suggesting that it was unlikely that they were having inverse agonist effects at CB_1_ receptors or signaling via G_s_-coupled receptors such as GPR119 or GPR6. However, given that there is evidence of biased agonist activity by cannabinoids [53] and we have shown that both ligands significantly elevated IP_1_ production in isolated mouse and human islets we cannot rule out SR141716A and/or AM251 signalling through a nominally G_s_-coupled receptor via G_q_-biased signalling. The elevation in IP_1_ implies GPR55-independent, G_q-_coupled receptor signalling by SR141716A and AM251 in islets and further studies using inhibitors of G_q_ and PLC are required to confirm this mechanism of action in islets. Possible G_q_-coupled candidates are GPR18 or GPR92, both of which are phylogenetically closely related to GPR55 [50] and activated by some cannabinoids [50, 51]. It has been reported that GPR18 and GPR92 activation is associated with transient elevation of [Ca^2+^]_i_ [50, 54], consistent with our IP_1_ data, although nothing is known about the functional role of these receptors in islets. We did not detect *GPR18* mRNA in human islets [55], so this receptor cannot be responsible for our observations of increased IP_1_ generation in human islets in response to SR141716A and AM251. GPR92 is a plausible candidate mediating the effects of SR141716A and AM251 in islets, and its upregulation following GPR55 deletion could be responsible for the elevated insulin secretory response to SR141716A that was observed in *Gpr55*^*−/−*^ islets. Further study in this area is dependent on the availability of GPR92-selective antagonists and studies in islets isolated from *Lpar5*^*−/−*^ mice.

Our qPCR analysis also indicated that *Trpv1* mRNA was upregulated 15.3 ± 4.3-fold in islets from *Gpr55*^*−/−*^ mice and it is possible that TRPV1 activation by SR141716A and AM251 was responsible, at least in part, for the stimulatory effects that we observed in islets following GPR55 deletion. Activation of this cation channel by capsaicin is coupled to TRPV1-dependent stimulation of calcium in INS-1E β-cells [56] and insulin secretion in mice [57, 58]. However, while capsaicin also stimulates insulin secretion in minced pancreas samples [57] and RIN insulinoma cells [58] it was without effect on non-selective cationic currents in primary rat β-cells [59] and failed to increase calcium in primary rat and human β-cells [56]. There is no information to date on the effects of SR141716A and AM251 via TRPV1 in islets, but as *Trpv1*^*−/−*^ mice are available for research future studies should be directed to determine whether stimulation by these ligands is reduced or abolished in islets isolated from these mice.

We have previously reported that LH-21 protected mouse and human islets from apoptosis in vitro through a GPR55-dependent mechanism [4] and had anti-inflammatory and cytoprotective effects on islets when administered in vivo [60], while exposure to CB_1_ and CB_2_ agonists did not affect mouse or human islet apoptosis [61, 62]. Conversely, the endocannabinoid system has been implicated in mediating increased islet apoptosis [63, 64]. In the current study we showed that SR141716A and AM251 have direct anti-apoptotic effects in isolated mouse and human islets and the use of islets from *Gpr55*^*−/−*^ mice indicated that, as for stimulation of insulin secretion, and IP_1_ generation, this was through a GPR55-independent cascade. Upregulation of CB_1_ receptors in islets from *Gpr55*^*−/−*^ mice could contribute to the anti-apoptotic effects of the cannabinoid ligands in these islets since JD5037, a CB_1_ receptor inverse agonist, reduced TUNEL-positive cells in islets [65].

Both ligands also stimulated human β-cell proliferation, but SR141716A abolished and AM251 reduced the low level of mouse β-cell proliferation. The reasons underlying these differences in effects of SR141716A and AM251 on β-cell proliferation in human and mouse islets are not known, but it is possible that they were related to the islet sources: islets were isolated from lean, male WT and *Gpr55*^*−/−*^ mice, whereas the human islets were from obese, female donors (BMI of 28.9 ± 0.96), where β-cell expansion capacity is enhanced [66]. Our availability of islets from normal weight donors was not sufficient for us to directly compare β-cell proliferation in lean populations of mouse and human islets, to determine whether the stimulatory effects of SR141716A and AM251 were indeed secondary to the islets having been obtained from obese donors. Alternatively, the differences may reflect species-dependent variations since anti-proliferative effects of SR141716A and AM251 have previously been reported in mouse preadipocytes [67] and mouse olfactory epithelium [68], consistent with our observations. We observed enhanced human β-cell proliferation in islets from three different donors, and it is possible that activation of GPR92 in human islets mediates this stimulatory effect on proliferation, as it does in human keratinocytes [69, 70]. SR141716A and AM251 also significantly increased human islet area and the number of β-cells per islet: it is unlikely that human islet β-cell proliferation fully accounts for the increases in these parameters given the very small increase in proliferation in response to SR141716A and AM251 (< 1 Ki67^+^ β-cell per islet). Therefore, since we observed that the ligands decreased stimulated human islet apoptosis the most likely explanation for increased human islet area and β-cell number following rimonabant and AM251 treatment is that these ligands protected against β-cell apoptosis induced by maintenance of 250 islets in culture without medium change for 48 h, consistent with the protective effects of GPR55 agonists and CB_1_ antagonists against human and mouse islet apoptosis that have been previously reported [4, 65].

In summary, our work provides the first evidence that SR141716A and AM251 are not GPR55 agonists in islets, as their effects are maintained in islets from *Gpr55*^*−/−*^ mice. Our observations of stimulation of insulin secretion and human β-cell proliferation, and protection against apoptosis in vitro*,* support SR141716A and AM251 having direct beneficial effects on islet function. However, their ability to induce insulin release from human islets at sub-stimulatory glucose concentrations contra-indicates against their use for treating type 2 diabetes as this could lead to hypoglycaemia in vivo. Additionally, our qPCR data showing that deletion of *Gpr55* promotes upregulation of *Cnr1, Lpar5* and *Trpv1,* and downregulation of *Gpr119* suggest a potential cross-regulation between GPR55 and other cannabinoid receptors in islets that warrants further research.

### Electronic supplementary material

Below is the link to the electronic supplementary material.

Supplementary file1 (DOCX 353 kb)

## Abbreviations

AM251: 1-(2,4-Dichlorophenyl)-5-(4-iodophenyl)-4-methyl-*N*-(piperidin-1-yl)-1H-pyrazole-3-carboxamide
BMI: Body Mass Index
Bp: Base pair
BrdU: Bromodeoxyuridine (5-bromo-2′-deoxyuridine)
Cch: Carbachol
CB_1_: Canonical cannabinoid receptor type 1
CB_2_: Canonical cannabinoid receptor type 2
CMRL: Connaught Medical Research Laboratories
Ct: Cycle threshold
DAPI: 4′,6-Diamidino-2-phenylindole
GLP-1: Glucagon-like peptide-1
GPR55: G-protein-coupled receptor 55
G_q_: G_q_ alpha subunit
G_s_: G_s_ alpha subunit
HTRF: Homogeneous time resolved fluorescence
IP_**1**_: Inositol-1-phosphate
IP_3_: Inositol trisphosphate
LH-21: 5-(4-Chlorophenyl)-1-(2,4-dichlorophenyl)-3-hexyl-1H-1,2,4-triazole
M_3_: Muscarinic receptors
RPMI: Roswell Park Memorial Institute
SR141716A (rimonabant): 5-(4-Chlorophenyl)-1-(2,4-dichloro-phenyl)-4-methyl-*N*-(piperidin-1-yl)-1H-pyrazole-3-carboxamide
