# Supplementary material for: The cannabinoid ligands SR141716A and AM251 enhance human and mouse islet function via GPR55-independent signalling
Source: Cell Mol Life Sci. 2020 Jan 10;77(22):4709–23. doi: 10.1007/s00018-019-03433-6 (PMC7599183; doi:10.1007/s00018-019-03433-6)
Supplement: Supplementary file 1 — Supplementary file1 (DOCX 353 kb) [file 18_2019_3433_MOESM1_ESM.docx]

SUPPLEMENTARY INFORMATION

**SUPPLEMENTARY FIGURE S1. Chemical structures of SR141716A and AM251.** SR141716A (A) and AM251 (B) are biarylpyrazole compounds. The p-chloro group at C-5 of the pyrazole ring in SR141716A is replaced by a p-iodo group in AM251.


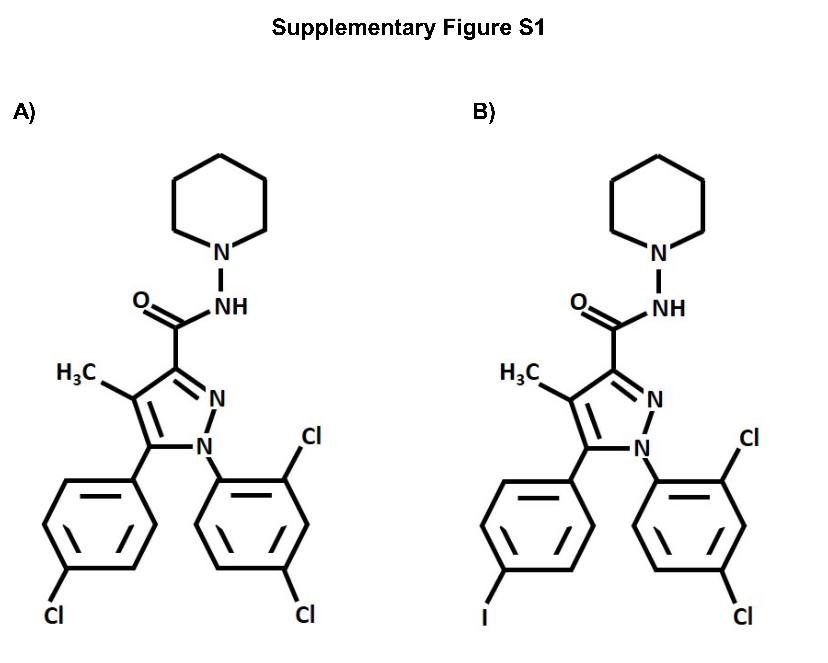


**SUPPLEMENTARY FIGURE S2**. **Agarose gel image.** Amplicons of the correct sizes for genotyping of *Gpr55^+/+^* (207 bp) and *Gpr55^−/−^* mice (299 bp).


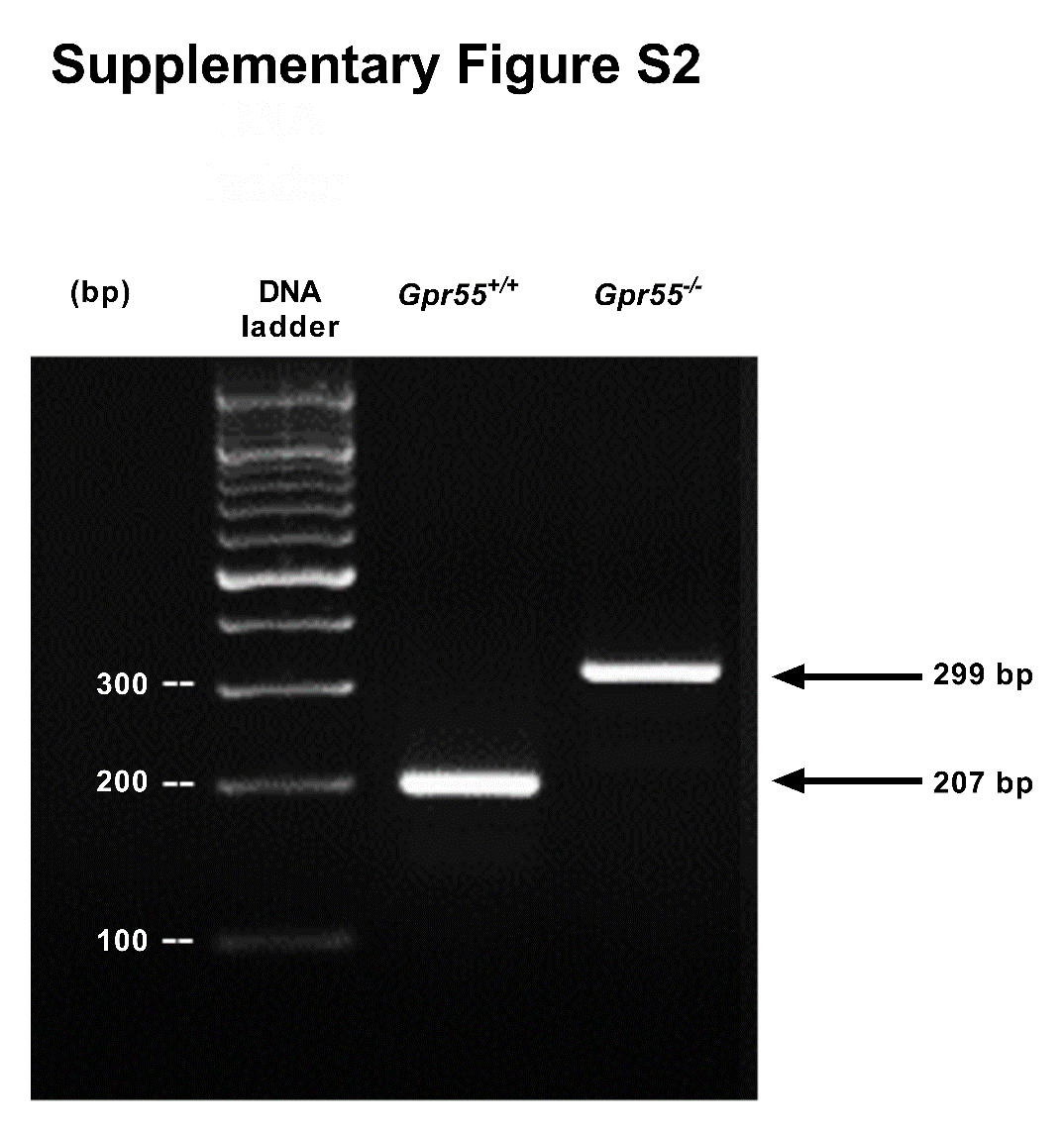


**SUPPLEMENTARY Table S1. Characteristics of the organ donors and the human islet preparations used in this study**.

| **Donor** | **Gender** | **Age**  **(years)** | **BMI**  **(kg/m^2^)** | **Islet purity (%)** | **Islet viability**  **(%)** |
| --- | --- | --- | --- | --- | --- |
| Number 1 | Female | 23 | 20 | 90 | 85 |
| Number 2 | Female | 43 | 30 | 65 | 75 |
| Number 3 | Female | 47 | 36 | 70 | 75 |
| Number 4 | Male | 34 | 26 | 75 | 75 |
| Number 5 | Male | 60 | 32 | 90 | 80 |
| Number 6 | Female | 52 | 23 | 75 | 80 |
| Number 7 | Female | 44 | 29 | 80 | 85 |
| Number 8 | Male | 40 | 24 | 45 | 90 |
| Number 9 | Male | 55 | 31 | 35 | 85 |
| Number 10 | Female | 37 | 30 | 90 | 85 |
| Number 11 | Female | 40 | 27 | 50 | 80 |
| Number 12 | Male | 58 | 29 | 70 | 70 |
| Number 13 | Female | 38 | 37 | 80 | 90 |
| Number 14 | Female | 53 | 23 | 85 | 60 |
| Number 15 | Male | 41 | 20 | 70 | 85 |
| Number 16 | Female | 55 | 24 | 80 | 85 |
| Number 17 | Male | 50 | 24 | 80 | 85 |

**SUPPLEMENTARY Table S2. Primers used for quantitative RT-PCR.** Mouse and human primers from Qiagen were used to quantify mRNAs encoding CB_1_, GPR119, GPR18, GPR92, OPRD1, TRPV1, GPR3, GPR6 and GPR12, and β-actin in islets isolated from *Gpr55^+/+^* and *Gpr55^−/−^* mice and in human islets.

| **Gene symbol** | **Primer assay**  **(*Mus musculus* and *Homo sapiens*)** |
| --- | --- |
| *Cnr1* | QT02522457 |
| *Gpr119* | QT01758953 |
| *Gpr18* | QT00129227 |
| *Lpar5* | QT00312571 |
| *Oprd1* | QT00103250 |
| *Trpv1* | QT00167048 |
| *Gpr3* | QT00249732 |
| *Gpr6* | QT00296044 |
| *Gpr12* | QT01062656 |
| *Actb* | QT00095242 |
| *CNR1* | QT00203287 |
| *GPR119* | QT00231609 |
| *GPR18* | QT01001504 |
| *LPAR5* | QT02449860 |
| *OPRD1* | QT00000210 |
| *TRPV1* | QT00046109 |
| *GPR3* | QT00203014 |
| *GPR6* | QT00202272 |
| *GPR12* | QT00205772 |
| *ACTB* | QT00095431 |

**SUPPLEMENTARY Table S3.** **List of primary and secondary antibodies used for immunofluorescence staining.**

| **Antibody** | **Dilution** | **Vendor** | **Catalogue number** |
| --- | --- | --- | --- |
| Anti-Ki67  (rabbit) | 1:200 | Abcam | ab15580 |
| Anti-insulin  (guinea pig) | 1:200 | Dako | A0564 |
| AlexaFluor 488  (goat anti-rabbit) | 1:150 | Jackson ImmunoResearch Laboratories | 111-545-003 |
| AlexaFluor 594  (donkey anti-guinea pig) | 1:150 | Jackson ImmunoResearch Laboratories | 711-586-152 |
